# Substance misuse teaching in undergraduate medical education

**DOI:** 10.1186/1472-6920-14-34

**Published:** 2014-02-17

**Authors:** Janine Carroll, Christine Goodair, Andrew Chaytor, Caitlin Notley, Hamid Ghodse, Peter Kopelman

**Affiliations:** 1 Psychology Department, University of Chester, Critchley Building, Parkgate Road, Chester, UK; 2 International Centre for Drug Policy, St George’s University of London, Cranmer Terrace, London, UK; 3 School of Medicine, Pharmacy and Health, Durham University, Queen’s Campus, Stockton-on-Tees, UK; 4 Norwich Medical School, University of East Anglia, Norwich, UK; 5 Chair National Steering Group, Principal, St George's University of London, London, UK

## Abstract

**Background:**

Over 12,000 hospital admissions in the UK result from substance misuse, therefore issues surrounding this need to be addressed early on in a doctor’s training to facilitate their interaction with this client group. Currently, undergraduate medical education includes teaching substance misuse issues, yet how this is formally integrated into the curriculum remains unclear.

**Methods:**

Semi-structured interviews with 17 key members of staff responsible for the whole or part of the undergraduate medical curriculum were conducted to identify the methods used to teach substance misuse. Using a previously devised toolkit, 19 curriculum co-ordinators then mapped the actual teaching sessions that addressed substance misuse learning objectives.

**Results:**

Substance misuse teaching was delivered primarily in psychiatry modules but learning objectives were also found in other areas such as primary care placements and problem-based learning. On average, 53 teaching sessions per medical school focused on bio-psycho-social models of addiction whereas only 23 sessions per medical school focused on professionalism, fitness to practice and students’ own health in relation to substance misuse. Many sessions addressed specific learning objectives relating to the clinical features of substance dependence whereas few focused on iatrogenic addiction.

**Conclusions:**

Substance misuse teaching is now inter-disciplinary and the frequent focus on clinical, psychological and social effects of substance misuse emphasises the bio-psycho-social approach underlying clinical practice. Some areas however are not frequently taught in the formal curriculum and these need to be addressed in future changes to medical education.

## Background

Substance misuse is a major public health issue in the UK, with 36.5% of 16 to 59 year old adults in England and Wales taking illicit drugs throughout their lifetime [1]. Furthermore, children as young as 11 years of age are smoking, drinking alcohol or taking drugs [2]. The impact this has on healthcare systems is profound; 12,344 reported hospital admissions in England were the result of drug poisoning with drug-related mental health and behavioural disorders also featuring in hospital admissions [1]. Currently, the Department of Health, UK, is addressing the issues related to substance misuse by focusing on the treatment of drug users and the supply of/demand for drugs [3]. However, substance misuse remains a major public health concern.

It is necessary to ensure health professionals are equipped with the skills required to help treat and manage people who misuse substances. General practitioners (GP) are often amongst the first health professionals to diagnose people with a substance misuse problem, yet these discussions between patient and GP often focus primarily on tobacco and alcohol use and can often be difficult for both participants to discuss [4]. This suggests that substance misuse problems can be missed by the doctor during consultations. Furthermore, additional complications may arise for the doctor if the patient is a colleague or other healthcare professional. Although doctors may have similar rates of alcohol misuse as the general population, their high use of prescription drugs as a form of self-treatment is widely recognised and colleagues can be unwilling to intervene if this use becomes problematic [5]. Additionally, substance misuse can have an impact on doctors’ fitness to continue practising in clinical environments. Addressing these issues at an early stage of medical training therefore is essential.

Substance misuse topics in medical education predominately focus on the effects of smoking or alcohol consumption with less information regarding legal and illegal drugs. O’Brien and Cullen found that despite increases in the prevalence of substance misuse, there had been no increase in the time allocated to teach undergraduate medical students substance misuse topics in Ireland [6]. An average of 6 hours of formal substance misuse training has been reported by medical schools in the UK and is mainly conducted within psychiatry and public health modules [7,8]. However, this does not account for the learning that occurs in the informal teaching environment or via the hidden curriculum, both of which are influential in shaping students’ learning and subsequent interaction with patients [9]. Since medical students have unique experiences influenced by their informal learning, it is important that medical education addresses the disparity between the amount of formal teaching and future clinical exposure to substance misuse issues to ensure that all students are provided with some formal education in this area.

Recommendations for core medical competencies on alcohol and other drugs have been made for postgraduate curricula for all doctors. A working group from the medical Royal Colleges published a report in 2012 which suggested that postgraduate medical curricula should consider incorporating learning objectives such as identifying the effects of using drugs, identifying the long-term implications of using drugs and being able to become confident to address drug use with their patients [10]. Some of these recommendations may occur within more informal learning experiences, such as direct contact with patients with drug problems [11] or through special study components (SSC). It is essential however that these recommendations become embedded within the formal undergraduate and postgraduate curricula in order for all students to cover the learning objectives and not just for those who happen to have relevant informal learning experiences.

This paper describes a national project run over two phases. Phase one (2005–2007) involved all medical schools in the UK to develop consensus guidance on the integration of teaching about alcohol, drugs and tobacco in the undergraduate medical curricula. Phase two (2008–2011) focused on the implementation of the guidance in to the curricula of English medical schools. In the first phase a survey was undertaken to gather information about substance misuse teaching and learning. The second phase focused on mapping substance misuse teaching within English medical schools thus identifying perceived and actual teaching occurring in undergraduate medical curricula. The project did not focus on the informal teaching that can occur within medical education nor the number of hours dedicated to the teaching of substance misuse but aimed to identify the number of actual teaching sessions that had formal learning objectives relating to substance misuse.

## Methods

The project was conducted in two phases. Phase one (2005–2007) involved conducting semi-structured interviews with key people. The questions asked were informed by a literature review of substance misuse teaching in undergraduate medical education and discussions with experts in the area. The potential interviewees within each UK medical school were asked if they would consent to participate in the semi-structured interview to identify their perceptions of the substance misuse teaching that occurred in their respective medical schools. The responses from these interviews facilitated the development of a substance misuse toolkit which focused on the specific learning objectives generally devised for substance misuse teaching. Phase two involved appointed curriculum co-ordinators mapping the actual teaching and learning of substance misuse using the toolkit devised in phase one and more on this has been described in another paper [12]. The second phase however identified the actual number of teaching sessions that were delivered which will be reported here.

### Participants

Participants in phase one were either the head of the undergraduate medical curriculum (such as the Director of the medical school) or other key people who were responsible for the major component of teaching about substance misuse, usually the organiser of the Psychiatry module. Two researchers identified and contacted potential participants via email or telephone call and 19 participants were recruited (two provided written feedback). Participants in phase two were the English medical schools who appointed a curriculum co-ordinator to map the areas of substance misuse teaching.

## Materials

A semi-structured interview was designed during discussions held by specialists forming an expert panel and conducted with participants in the first phase of the project. The interview questions covered curriculum content, curriculum delivery, assessment, student selected components and learning materials. Example interview questions include “Could you describe how teaching and learning about substance misuse is organised?” (curriculum delivery) and “Are there Student Selected Components in the area of substance misuse available to students?” (student selected components). The responses were collated and used by a working group of healthcare professionals and educators to devise a UK consensus guidance document on substance misuse teaching in the undergraduate medical curriculum. To accompany this document, a substance misuse toolkit was devised that set out the core aims and learning outcomes for undergraduate medical curricula. The toolkit focused on bio-psycho-social models of addiction, professionalism and self-care, clinical assessments of patients, treatment interventions, specific disease and speciality topics and epidemiology, public health and society.

### Procedure

In phase one, two researchers identified and arranged semi-structured telephone interviews with consenting key staff over a period of 15 weeks in 2005 to obtain feedback on substance misuse teaching. The responses were then collated and the key aspects were identified by the two researchers. Using these responses, a toolkit was used by curriculum co-ordinators in phase two to identify the exact teaching sessions that featured learning objectives relating to substance misuse. Curriculum co-ordinators began collecting this information at different time-points over a 12-month period between 2009 and 2011. Due to the different data collection time-points, some of the information collated during the mapping process was not available for the deadline of writing reports concerning the project but has since been submitted and is reported in this paper.

### Ethical approval

This project was a review of the current teaching and learning that occurs within undergraduate medical education. All work was conducted based on the Declaration of Helsinki ethical principles for research involving human participants. No formal ethical approval was required from an ethics committee as the interviews were providing feedback on the medical course and the toolkit was identifying teaching sessions only that covered the different substance misuse learning objectives.

## Results

### Demographic statistics

Out of 32 UK medical schools, 22 interviews were conducted and ten medical schools provided written responses to the questions, resulting in feedback being obtained from all medical schools. During phase two, 18 out of the 19 English medical schools (a 95% response rate) submitted data from the mapping process.

### Phase one

Participants reported substance misuse learning objectives and outcomes existed at the programme level, the module level and the individual teaching session level. Although they acknowledged explicit reference to substance misuse at the programme level might be limited, specific reference was mentioned in relation to substance misuse and self-care while implicit reference was made to the development of an appropriate attitude to patients. The majority of teaching and learning about substance misuse were reported to be located within psychiatry modules, however other disciplines were also perceived to include teaching on substance misuse. Primary care and general practice attachments were mentioned by nine participants as being one area where students received substance misuse teaching, and cross-year teaching such as communication skills, problem-based learning (PBL), and community based medicine (n = 9) were also reported to provide substance misuse learning objectives. Five participants reported that substance misuse teaching was delivered during public health modules while eight stated that these issues were raised directly (and indirectly) in personal and professional modules or introductory courses. When asked specifically about student selected components (SSC), eleven medical schools reported offering at least one SSC focusing on substance misuse. However, one participant described how nearly one-third of SSC topics offered in their curriculum were related to substance misuse, suggesting variation amongst medical schools concerning the number of substance misuse SSCs. As the aim of this phase was not to identify the number of hours spent formally teaching substance misuse issues, participants only reported where in the curriculum substance misuse was formally taught.

A range of learning materials were used to support substance misuse teaching including CD-ROMs, online resources, handouts, powerpoint presentations, alcohol information packs and an internet game focusing on managing alcoholic patients. Assessing students’ knowledge of these issues was mainly conducted using Observed Structured Clinical Examination stations (n = 10) and predominately focused on assessing students’ communication skills when interacting with patients with substance misuse problems.

### Phase two

Analysis of the data gathered during the mapping process revealed that bio-psycho-social models of addiction had the most number of teaching sessions identified (n = 958, an average of 53 per medical school), with professionalism and self-care having the least number of teaching sessions (n = 418, an average of 23 per medical school; Table 1). These figures are higher than those reported elsewhere [12] due to some of the information becoming available after the initial report of the project had been written.

**Table 1:** The number and average of substance misuse teaching sessions identified in the general medical curriculum

**Learning outcomes area**	**Number of teaching sessions**	**Averaged (per school)**
Bio-psycho-social models of addiction	958	53
Professionalism and self-care	418	23
Clinical assessment of patients	942	52
Treatment interventions	921	51
Epidemiology, public health and society	578	32
Specific disease and speciality topics	846	47

Within the bio-psycho-social models of addiction theme, defining substance misuse, dependence and addictive behaviour was the most frequently taught topic in medical education (n = 291, an average of 16 sessions per medical school). Demonstrating awareness of the causes of addiction and describing the chemical absorption and excretion of addictive drugs were the least frequently covered topic (n = 7 sessions per medical school; Table 2).

**Table 2:** The actual number and average of teaching sessions per medical school identified by curriculum co-ordinators

**Theme in toolkit**	**Learning outcome**	**Number of formal teaching sessions with substance misuse learning objectives**	**Averaged (per medical school)**
Bio-psycho-social models of addiction	1a. Define substance misuse, dependence and addictive behaviour and distinguish between acceptable and problematic use	291	16
	1b. Demonstrate awareness of the range of substances that can be misused, the different types and classes of addictive substances, their alternative and colloquial names and their effects	143	7
	1c. Demonstrate awareness of the psychological, social, biological and genetic causes of dependence and addiction, the interactions between such factors in the individual and the different models used to describe addiction	157	8
	1d. Describe the absorption, distribution, excretion and metabolism of drugs of addiction	133	7
	1e. Describe the physical effects of addiction, including the key effects of drug addiction on neurotransmitter systems, mechanisms of drug tolerance and the physiological effects of withdrawal	234	13
Professionalism, fitness to practice and students’ own health	2a. Demonstrate a professional attitude towards substance misusers which incorporates a non-judgemental approach and respect for a patient's autonomy	109	6
	2b. Describe the risk factors for substance misuse in themselves, in medical students and in healthcare workers	70	3
	2c. Describe the sources of help for students and doctors with drug and alcohol related problems	35	1
	2d. Describe how substance misuse problems may affect a healthcare professional’s judgement, performance and care for their patients	59	3
	2e. Describe the need to balance due concern for the health of a colleague with responsibilities for the safety and welfare of patients	56	3
	2f. Outline the role of the GMC and medical schools in ensuring students and doctors’ fitness to practice	59	3
	2g. Demonstrate understanding of iatrogenic addiction	30	1
Clinical assessment of patients	3a. List the major clinical features of alcohol and drug dependence	263	14
	3b. Describe the range of clinical outcomes of addiction and discuss the prognosis and management	154	8
	3c. Take a focussed drug and alcohol history	134	7
	3d. Elicit signs of alcohol or drug misuse through physical and mental state examinations and identify and prioritise medical and psychosocial problems associated with substance misuse	136	7
	3e. Demonstrate appropriate skills for communicating sensitively with patients about substance misuse issues and for dealing with difficult, aggressive or intoxicated patients, balancing assessment need with their own safety and that of others	105	5
	3f. Appropriately order and interpret urine and blood screening tests for drugs of addiction, use standardised screening and assessment instruments to detect alcohol and drug levels and describe other special investigations and how to interpret results	90	5
	3g. Carry out a psychological assessment of a patient’s readiness to implement change	60	3
Treatment interventions	4a. Describe the basic treatment regimens for various addictions and withdrawal states	134	7
	4b. Describe the basis of commonly used therapies for addiction, such as Brief Intervention therapy	66	3
	4c. Describe the variety of UK agencies to which patients with addiction problems can be referred and how and where to make appropriate referrals for treatment	46	2
	4d. Advise a patient on risk-reduction strategies for drug use	75	4
	4e. Demonstrate awareness of risk related to needle use and disposal for healthcare workers and patients	33	1
	4f. Advise a patient appropriately on reducing or abstaining from drinking and smoking and implement a treatment plan with the patient	319	17
	4g. Advise addicted women on how to stabilise/discontinue substance use to minimise impact on foetal and maternal health	48	2
	4h. Demonstrate awareness of the need to assess patients’ capacity to consent to treatment	159	8
	4i. Describe the impact of substance misuse on concordance with treatment including Discharge Against Medical Advice and drug interactions	41	2
Epidemiology, public health and society	5a. Describe UK policies on drug use, drug dispensing and prescribing and on alcohol and smoking	67	3
	5b. Describe UK legislation on controlling drugs, alcohol and tobacco, including the legal limits for alcohol and driving and the recommended maximum limits for alcohol consumption	76	4
	5c. Describe UK strategies for the prevention of drug misuse	26	1
	5d. Describe international policies and strategies to limit drug supply and demand	10	0.5
	5e. Describe the epidemiology of alcohol consumption, smoking, drug misuse in the general population and specifically in doctors and other healthcare professionals	97	5
	5f. Describe the problems associated with self-medication	36	2
	5g. Demonstrate awareness of the risks in different work environments and the need for employers to have drug and alcohol policies	23	1
	5h. Describe the effects of addiction on individuals, their families, friends and colleagues in a range of age-groups; from children and adolescents to older people	69	3
	5i. Describe the long-term social consequences of various types of addiction and substance misuse, including the economic consequences and the links between crime and substance misuse	63	3
	5j. Describe the risks to the children of addicted parents including child protection policies and a doctor’s duty to implement these	59	3
	5k. Demonstrate an understanding of the principles of rational prescribing and the use of psycho-active medication	52	2
Specific disease and speciality topics	6a. Recognising life-threatening complications of substance misuse, including septicaemia, pulmonary emboli and overdose and be able to carry out appropriate interventions	152	8
	6b. Describe and explain the links between substance misuse and accidents; lung disease, specifically smoking; anxiety, depression, dementia, schizophrenia; self-harm and suicide; heart disease and hypertension; liver disease, pancreatitis and gastritis; infectious diseases, including HIV and hepatitis B and C; cancers; sleep disorders; weight problems	584	32
	6c. Show awareness of substance misuse in the aetiology of neurological conditions including seizures, par aesthesia and stroke	52	3
	6d. Describe the effects of addiction, drug and alcohol use on pregnancy	58	3

For the professionalism and self-care theme, the topic of demonstrating a professional attitude was frequently focused upon (n = 109, an average of 6 sessions per medical school) whereas demonstrating an understanding of iatrogenic addiction was the least frequently covered topic (n = 30, an average of 1 session per medical school). When mapping the teaching that occurs around the clinical assessment of patients with substance misuse problems, co-ordinators identified 263 teaching sessions (an average of 14 sessions per medical school) covering the major clinical features of alcohol and drug dependence. Yet only 60 sessions (an average of 3 sessions per medical school) focused on conducting a psychological assessment of a patients’ readiness to implement change.

Table 2 also shows variation in the number of teaching sessions focusing on the treatment and interventions available to people who misuse substances, with the majority focusing on how to advise a patient appropriately on reducing or abstaining from using alcohol or tobacco (n = 319, an average of 17 sessions per medical school). The least frequently taught topic in this area related to students ability to demonstrate their awareness of risk related to needle use and disposal (n = 33, an average of 1 session per medical school). Within the epidemiology, public health and society theme, learning about the distribution and determinants of alcohol consumption, smoking and drug use in both the general and the medical population was the most frequently taught (n = 97, an average of 5 sessions per medical school). In contrast, curriculum co-ordinators found little teaching covered the international policies and strategies in place to limit the supply and demand of drugs (n = 10, an average of half a teaching session per medical school).

Finally, many teaching sessions were offered that addressed specific substance misuse topics such as describing the link between substance misuse and accidents, lung disease or mental health (n = 584, an average of 32 sessions per medical school). Few sessions however focused on making students aware of how substance misuse can influence the aetiology of neurological conditions (n = 52) and the effects of addiction and drug consumption during pregnancy (n = 58), an average of 3 sessions for both learning objectives per medical school.

## Discussion

The above mapping process revealed the areas of substance misuse teaching and learning that were perceived to be and were actually covered within medical education. Changes have been documented in relation to how substance misuse is incorporated into the curriculum [13], and the increase in specific learning objectives specialising in linking substance misuse to health issues could reflect an increase in the number of core and elective modules (such as SSCs, clinical skills, communication skills etc.) that now include some reference to patients who misuse substances. Furthermore, the rise in research relating to substance misuse has provided a comprehensive evidence base for teaching students good medical practice [14,15] and has enabled medical education to develop both generic and specific substance misuse learning objectives.

The most prevalent area covered in the undergraduate curriculum was bio-psycho-social models of addiction. The clinical, psychological and social impact of drug misuse and dependence has long been accepted as important for medical students to consider when interacting with patients and colleagues. Clinical assessment and treatment interventions are covered comprehensively suggesting students are now being given the opportunity to develop the skills required for interacting with substance misuse in patients. Students have been found to ask about drug use as a prevention strategy during these formal clinical assessments [16], suggesting an awareness early on in their education of how substances can be used to prevent as well as treat medical conditions. Identifying the clinical features of substance dependence is also an important area for students as many begin their careers in general practice where patients misusing prescription drugs can be a problem [17], therefore the clinical knowledge acquired around the features, physical effects and treatment strategies of substance misuse is essential. Additionally, substance misuse learning objectives that feature within different modules support the view of educators that these issues are located in various modules across all years of medical education.

Few teaching sessions focused on professionalism, fitness to practice and students’ own health in relation to substance misuse. One area that was emphasised focused on developing a professional attitude towards patients who misuse substances – a positive finding, as formal teaching programmes that provide access to substance misusers have been shown to effectively improve medical students’ attitudes towards this patient group [11]. However, there was a lack of teaching focusing on understanding iatrogenic addiction. The use of analgesic drugs such as morphine are often effective in reducing pain yet despite doctors’ propensity to administer these drugs confidently in clinical environments, they often have addictive properties and may contribute to the development of iatrogenic illness in patients [18]. Reasons for the lack of understanding regarding iatrogenic addiction include different perceptions of doctors, nurses and patients regarding pain intensity, a belief that patients exaggerate their pain in order to obtain more medication [19] and an underestimation by doctors of the potential effect of drugs such as sedatives in promoting dependence or addictive behaviour in patients [20]. It is important therefore to raise awareness of the effects of iatrogenic addiction in all health professionals, and this could be integrated into the undergraduate medical curriculum through SSCs, PBL, and other formal teaching sessions supporting a bottom-up approach to learning [21].

There are however some limitations to this project which need to be addressed. First, the project did not focus on the learning that can occur within informal experiences or via the hidden curriculum, both of which are important sources of learning for medical students [9] and it is unclear what informal learning occurs in relation to substance misuse. Secondly, the data was only collected from English medical schools and did not cover the curriculum delivered in other parts of the UK. Thirdly, it is unclear whether the mapping conducted by the curriculum co-ordinators was consistent across all participating medical schools and there were no external checks to identify the accuracy of the mapping process. Lastly, a formal evaluation of the project was not performed but would have been beneficial to appraise the effectiveness of conducting this type of research. Future projects would therefore benefit from addressing these limitations and investigating the impact of informal learning experiences on medical students’ knowledge and understanding of substance misuse issues. Furthermore, future projects of this design would benefit from including an evaluation process and internal reliability checks before conducting it in other medical schools across the UK.

Despite these limitations, one result of the curriculum mapping process is that co-ordinators were able to implement a variety of changes such as re-writing PBL learning outcomes to specifically include substance misuse, updating library and internet resources, and introducing lectures and workshops targeting substance misuse issues. For example, one medical school wrote a policy in relation to drugs, alcohol and substance misuse which outlined the regulations and code of conduct for students to follow during their medical training. This was written in all module handbooks ensuring students’ awareness of the importance of substance misuse issues. Some medical schools worked with external agencies to increase students’ access to people who have substance misuse problems, initiatives that students reported as being interesting and conducive to their learning. In addition to individual changes being implemented at the different medical schools, substance misuse factsheets were generated to facilitate students’ learning in relation to specific issues (e.g. substance misuse and anaesthesia, substance misuse and pregnancy, substance misuse in gastroenterology). These could be accessed and used by all participating medical schools and focused on a wide range of areas in which substance misuse issues could arise. They were devised by a group of experts in the field to ensure that each help pertinent information relating specifically to each topic and were used by some medical schools to facilitate students’ learning.

A key question now, given the success of the project, is how best to sustain the positive changes implemented in the teaching of substance misuse to our future doctors, so that future graduating medical students continue to be better equipped to deal with substance misuse and to meet the requirements specified by the GMC. This project across both phases has enhanced the training and education of student doctors and has established a solid basis for substance misuse teaching through the implementation of UK corporate guidance and improvements in the quality of training of future doctors in English medical schools. Funding for a third phase has been found and the aims of this phase are to further develop and build on the existing network of academic champions/lead person within the medical schools to work on embedding substance misuse in to teaching through the corporate curricula and learning materials so as to ensure that the changes implemented in the teaching of substance misuse to our future doctors continue to be better equipped to deal with substance misuse.

## Conclusions

To conclude, this project has identified the areas and frequency of formal substance misuse teaching within the undergraduate medical education. The bio-psycho-social models of addiction are most frequently covered by medical schools allowing students to focus on the clinical aspects of substance misuse. However, one area that is not frequently covered in the formal curriculum is iatrogenic addiction and it is unclear whether this and other substance misuse issues are addressed during informal learning experiences. Future research therefore could investigate the influence of informal learning experiences in relation to substance misuse as well as how the positive teaching that currently occurs can be sustained to ensure graduating medical students are better equipped with the skills needed to deal with these issues in their future practice.

## Competing interests

The authors declare that they have no competing interests in relation to this article.

## Authors’ contributions

JC, CG, AC and CN made substantial contributions to the acquisition and interpretation of the data, have been involved in drafting and revising of the manuscript, and have given final approval of the version to be published. CG, HG and PK made substantial contributions to the conception and design of the project. HG and PK have been involved in revising the manuscript and PK has given final approval of the version to be published.

## Pre-publication history

The pre-publication history for this paper can be accessed here:

http://www.biomedcentral.com/1472-6920/14/34/prepub
